# Compensability of an enhanced incidence of spermatozoa with cytoplasmic droplets in boar semen for use in artificial insemination: a single cell approach

**DOI:** 10.1038/s41598-022-26020-5

**Published:** 2022-12-17

**Authors:** Heiko Henning, Anne-Marie Luther, Lisa Höfner-Schmiing, Dagmar Waberski

**Affiliations:** 1 Institute of Farm Animal Genetics, Friedrich-Loeffler-Institut, Neustadt am Rübenberge, Germany; 2 Unit for Reproductive Medicine / Clinic for Pigs and Small Ruminants, University of Veterinary Medicine Hannover, Foundation, Hannover, Germany

**Keywords:** Biotechnology, Cell biology

## Abstract

This single cell study aimed to clarify whether an elevated incidence of sperm with a retained cytoplasmic droplet (CD) can be compensated by a higher sperm number in boar semen doses to maintain fertility. Cluster analysis of motile spermatozoa (ten boars) revealed that spermatozoa with a CD are underrepresented in the fast, linearly moving sperm cohort compared to morphologically normal sperm. Nonetheless, the response to the motility stimulator procaine was barely affected in spermatozoa with distal CD (Cramer’s V = 0.14), but moderately affected in sperm with proximal CD (V = 0.28). Viability was lower in sperm with distal CD (*p* < 0.05) but not with proximal CD compared to normal sperm during 168 h storage of extended semen samples (n = 11) and subsequent thermic stress. Morphologically normal sperm from normospermic samples (n = 10) or samples with a high incidence (≥ 15%) of sperm with CD (n = 9) had similar motility patterns and responses to procaine. The origin of morphologically normal sperm had no effect on sperm viability (*p* > 0.05; n = 26). In conclusion, a moderately enhanced prevalence of sperm with CD seems to be compensable by an increase in sperm numbers in boar semen doses.

## Introduction

Artificial insemination (AI) in pigs is the most common and successful assisted reproductive technology in animal husbandry. The efficient production of semen doses with a high fertilisation capacity is a major challenge in modern pig breeding^1^. Selection of boars for genetic traits and elimination of low-quality semen prior to processing to insemination doses greatly contribute to production safety and efficiency in AI centres^2^. Based on numerous reports on the association of sperm quality and fertility^2–7^, pig breeding organisations have defined minimal requirements for relevant sperm traits, especially considering sperm motility and morphology^1^. A major reason for excluding semen from the processing to insemination doses is the presence of sperm with retained cytoplasmic droplets (CD), located either at the sperm neck (proximal position) or at the lower midpiece tail (distal position)^3,4^. Higher amounts of CD bearing sperm have been associated with reduced pregnancy rates, farrowing rates and smaller litter sizes^3,5–7^. Cytoplasmic droplets present a remnant of germ cell cytoplasm which physiologically migrates along the midpiece from the sperm neck to the annulus during epididymal transport^8^ and, in boars, are shed upon contact with seminal fluids within 1 min after ejaculation^9^. Although zootechnical and boar health management are well controlled in AI centres, a considerable number of boars permanently or transiently display an enhanced prevalence of sperm with CD in the ejaculate.

This situation occurs especially in the late summer season because of periods with a high temperature-humidity index and a decreasing photoperiod^3^. Consequently, boars are excluded from the production of semen doses if maximum acceptable values for CD, commonly set between 15 and 30%^1^, are exceeded. This pervasive situation does not only deprive the sow breeders from boars with the desired genetics but also affects the economics of pig breeding.

A possible solution could be proposed if spermatozoa with CD can be proven to be an undesired but compensable semen trait. Sperm with compensable defects are incapable of reaching the fertilisation site or interacting with the oocyte, whereas sperm with uncompensable defects (e. g. chromatin defects) are not selected by the female genital tract, and thus interact with oocytes but are not able to complete fertilisation or to sustain early embryogenesis^10,11^. In case of compensable defects, the total number of spermatozoa in the insemination dose could be increased in order to raise the number of morphologically intact spermatozoa to a “sufficient” level so that full fertility potential in the females is reached. Whether this option exists to counteract the impact of sperm with retained CD on the insemination outcome is currently unknown.

There are indications that a CD could be assigned to the category of compensable sperm defects which prevent such spermatozoa from fertilising. For example, in vivo recovery of spermatozoa from the uterotubal junction in inseminated pigs^12^ or *post coitum* of cervical mucus in humans^13^ showed a very low incidence of CD compared to ejaculated sperm. It was speculated that the negative selection of CD-bearing sperm in the female tract results from a reduced progressive motility, caused either by flagellar angulation or by a disturbed volume regulation due to accumulated osmolytes in the CD^14^. Alternatively, or additionally, the presence of CD could affect the response to female activation signals and thereby hinder sperm from arrival in the oviduct. Furthermore, sperm with CD do not bind to cultured porcine oviductal epithelial cells in vitro^15^ and to the zona pellucida in bovines^16,17^, thus precluding sperm with this defect from establishing the oviductal sperm reservoir and fertilising, probably caused by disturbed plasma membrane.

Sperm migration to the oviduct and formation of the oviductal sperm reservoir require undisturbed motility, an intact plasma membrane, and proper cell function. So far, knowledge of kinematics and plasma membrane integrity (viability) of sperm with CD at single cell level is lacking. Such observations would help to better understand the reason for fertilisation failure and to clarify the option to compensate for CD by an increase in sperm numbers in the insemination portion. Real-time single cell tracking of kinematics and identification of CD and other most prevalent sperm tail defects have recently become available in a new generation of computer-assisted semen analysis (CASA) systems^18^. Using this technology, in the present study, kinematics in boar spermatozoa with and without retained CD were analysed at a single cell level. Additionally, the responsiveness to a motility stimulator was examined to test whether the presence of a CD hampers the activation of sperm, which would indicate lower chances for arrival at the fertilisation site^19^. To clarify the possibility for compensation, we tested the hypothesis that kinematics and plasma membrane stability between morphological normal sperm from samples with low and high incidence of CDs do not differ. Overall, this study aims to estimate the potential to utilise genetics of boars with moderately elevated incidence of sperm with CD and thus to ensure AI efficiency without risking fertility loss.

## Material and methods

### Chemicals and reagents

All chemicals used were of analytical grade and, unless otherwise stated, purchased from Sigma-Aldrich (Steinheim, Germany), Merck (Darmstadt, Germany) or Roth (Karlsruhe, Germany).

### Semen samples

Boar semen samples with high or low incidence of morphological abnormal spermatozoa (MAS) were obtained during June and July 2021 from EU-admitted AI centres during their routine semen dose production. Animal care protocols were performed in accordance with ARRIVE guidelines^20^ and are in accordance with guidelines of the national animal welfare authorities. The experimental protocol was approved by the Animal Welfare Officer of the University of Veterinary Medicine Hannover, Foundation. Semen extended in Beltsville Thawing Solution from boars with an enhanced (≥ 15%) incidence of spermatozoa with CD and/or bent tails (high MAS samples) were sent from six AI centres upon request to the spermatology laboratory at the Unit for Reproductive Medicine, University of Veterinary Medicine Hannover, Foundation, Hannover, Germany. Semen samples from boars with a low (< 5%) prevalence of spermatozoa with CD and/or bent tails (low MAS samples) from the same AI centres were used as control. Semen tubes or bags were shipped overnight in insulated boxes at + 16 to + 18 °C and arrived at the laboratory within 24 h after semen collection. On the day of arrival, the percentage of motile sperm was evaluated with computer assisted semen analysis (CASA) and sperm morphology was evaluated in formaline citrate-fixed samples as described by Henning et al.^21^. Samples with a total motility below 50% or more than 5% morphological sperm defects other than CD or bent tails were not admitted to the study.

### Assessment of sperm kinematics and stimulation with procaine

Sperm kinematics were assessed in extended samples at 24 h after semen collection using the CASA system AndroVision® (Version 1.2, Minitüb, Tiefenbach, Germany) equipped with an automated, heated microscope stage, a digital camera (acA2440—75uc, Basler, Ahrensburg, Germany) and a TV adapter (60-C 1″ 1.0 × , Zeiss, Jena, Germany). Semen aliquots (5 mL) were incubated under air at 38 °C in a water bath for 30 min. Subsamples were placed in a 20 μL counting chamber (Leja, Products, Nieuw Vennep, the Netherlands) and at least 400 sperm/sample were recorded as described by Höfner, et al.^22^. Correct recognition of proximal CD, distal CD and bent tails by the algorithm for morphology classification was confirmed by visual assessment of sperm on the screen. Incorrectly tracked spermatozoa were excluded from the analysis. For the group of motile spermatozoa, curvilinear velocity (VCL), linearity (LIN), average amplitude of lateral head displacement (ALH) and beat cross frequency (BCF) were used as descriptors for kinematic clusters. Spermatozoa were considered as motile with values for VCL > 24 μm/s and ALH > 1 μm^22^. Sperm kinematics were also evaluated after stimulation with procaine^23^. To this end, aliquots of extended semen (6 mL) were centrifuged for 3 min at 3360 g. The supernatant was removed to a residual 0.5 mL and the pellet was resuspended in a modified non-capacitating Tyrode’s medium (96 mM NaCl, 20 mM HEPES, 5 mM glucose, 3.1 mM KCl, 0.4 mM, 0.4 mM MgSO_4_, 0.3 mM KH_2_PO_4_, 100 µg/mL gentamicin sulphate (SERVA, Heidelberg, Germany), 20 µg/mL phenol red, 1.0 mM sodium pyruvate, 21.7 mM sodium lactate) supplemented with procaine to yield a final concentration of 7.3 mM. A second pellet was resuspended in modified Tyrode’s solution lacking procaine and was used as control. Samples were incubated for 10 min at 38 °C under air in a water bath and thereafter analysed as described above.

### Assessment of sperm morphology and viability

Sperm morphology and viability (plasma membrane integrity) were assessed at a single cell level. For this, 100 µL of an aqueous solution containing 0.7% Eosin Y and 10% Nigrosin was added to 500 µL extended semen and incubated for 20 s at 38 °C in a metal block. Twenty µL of this solution was placed on a pre-warmed glass slide and a smear was produced using a second glass slide. Slides were then air-dried on a heat stage. Three hundred sperm per specimen were examined using a bright field optical microscope (Carl Zeiss, Göttingen, Germany) at × 1000 magnification with immersion oil. Positive eosin staining indicated sperm with a damaged plasma membrane (“non-viable” sperm).

## Experimental designs

### Experiment 1. Sperm kinematics

Single sperm data for motile spermatozoa from semen samples after a 30-min incubation period in BTS were pooled for 10 extended semen samples with ≥ 15% sperm with CD and/or bent tails (= high MAS samples; n = 10 boars) and nine semen samples with < 5% sperm with CD or bent tails (= low MAS samples; n = 9 boars). Individual spermatozoa were assigned to one of four morphology groups: (i) normal, (ii) proximal CD, (iii) distal CD or (iv) bent tail.

#### Experiment 1a): Impact of the presence of sperm with abnormal tail morphology (CD, bent tail) on motility of sperm with normal tails

It was tested whether the origin of morphologically normal sperm from samples with a high or low incidence of MAS influences their movement patterns. To this end, motile spermatozoa with normal morphology collected from the 10 high MAS semen samples and morphologically normal spermatozoa from nine low MAS semen samples were compared.

#### Experiment 1b): Impact of sperm tail morphology on sperm movement

To answer the question whether sperm tail morphology has a distinct impact on sperm movement patterns, the movement patterns of spermatozoa with a retained CD or bent tail were compared to morphologically normal sperm from the same sample.

### Experiment 2. Sperm response to procaine

Sperm response to the motility activator procaine was studied at a single cell level in the high MAS samples and low MAS samples. Kinematic patterns were recorded for the four tail morphology groups as described in Experiment 1.

#### Experiment 2a): Impact of the presence of sperm with abnormal tail morphology (CD, bent tail) on the movement patterns of sperm with normal tails after stimulation with procaine

It was tested whether the origin of morphologically normal sperm from high or low MAS samples influences their movement patterns in response to procaine.

#### Experiment 2b): Impact of sperm tail morphology on movement patterns after stimulation with procaine

The response to the motility stimulator procaine for spermatozoa with retained CD or bent tail was compared to morphologically normal sperm. For this, kinematic responses in the same samples were examined.

### Experiment 3. Sperm viability

The eosin-nigrosin staining technique^24^ was used to assess the presence of proximal CD, distal CD or bent tails concomitantly with cell viability. Semen samples with high CD (mean 23.2 ± 13.04; n = 15 boars) and samples with low CD (mean 2.0 ± 1.18; n = 11 boars) were examined after 24 h, 72 h, 144 h or 168 h storage at 17 °C and subsequent thermic incubation for 3 h at 38 °C.

#### Experiment 3a): Impact of the presence of sperm with abnormal tail morphology (CD, bent tail) on the viability of sperm with normal tails.

It was tested whether the origin of morphologically normal sperm from high or low MAS samples influences their viability during long-term semen storage and after subsequent thermic stress.

#### Experiment 3b): Impact of sperm tail morphology on sperm viability

The viability of spermatozoa with retained CD or bent tail was compared to morphologically normal sperm from the same samples.

### Statistical analysis

Cluster analysis was carried out using Excel (Microsoft Office 2010, Microsoft Corporation, WA, USA) and Statistical Analysis Software (SAS, version 9.4, Cary, NC, USA) essentially as described in Henning et al.^25,26^. In short, single sperm data (motility descriptors) from CASA were pooled for all samples after regular motility analysis (30-min incubation period in BTS extender; Dataset 1; n = 5439 sperm) or for all samples from the experiment on motility stimulation with procaine (Dataset 2; n = 8307 sperm). Eight motility descriptors were available from CASA analysis (VCL, VSL, VAP, LIN, STR, WOB, ALH and BCF). Parameters were correlated after testing for normality (PROC UNIVARIATE and PROC CORR). Whenever two or more parameters correlated ≥ 0.90, only one was chosen to enter the final clustering procedure to reduce the number of variables. For Dataset 1, the motility descriptors VCL, VSL, LIN, STR and BCF were chosen for the clustering procedure. For Dataset 2, all motility descriptors were used. The variables were standardised to a mean of 0 and a standard deviation of 1 to avoid any bias in the clustering procedure due to different parameter scales. Squared Euclidian distance as distance measure and the ‘centroid’ algorithm for cluster fusion were used for performing a hierarchical cluster analysis (PROC CLUSTER). The choice of a suitable solution from the clustering procedure was guided by the cubic clustering criterion (CCC), pseudo-F statistics and pseudo-t^2^ values. The aim of the exploratory data analysis was to obtain a solution that explained as much of the variance in the data set and contained as few as possible major clusters. A cluster was considered relevant when it contained at least 5% of sperm in any of the analysed subgroups (e.g. sperm with a bent tail). For Dataset 1, a solution with nine major clusters was chosen which explained 70.2% of the variance in the dataset. For Dataset 2, a solution with seven major clusters was chosen which explained 66.4% of the variance. To test whether distribution of sperm between the revealed cluster differed between normal spermatozoa originating from low MAS or high MAS samples or spermatozoa with different morphology, an ‘χ^2^’-test for homogeneity was performed (PROC FREQ). Cramer’s V (range 0 to 1) was used as a measure of the effect size that the origin of sperm or morphology had on the distribution of sperm to the different clusters. For interpretation, the following guidelines were followed as suggested by Cohen^27^: V < 0.10 = no effect, 0.10 < V ≤ 0.30 = small effect, 0.30 < V ≤ 0.50 = moderate effect, V > 0.50 = strong effect. The size of selected clusters of interest was compared between low and high MAS samples with Student’s t-test for unpaired samples, while changes in cluster size in a given set of samples due to procaine were tested for significance using the Student’s t-test for paired observations.

For viability data analysis, IBM SPSS Statistics Professional (SPSS Inc., IBM, Armonk, NY, USA) was used. Categorical variables were compared using the Chi-square test. In general, a *p*-value of less than 0.05 was considered statistically significant.

## Results

### Experiment 1: Sperm kinematics

#### Experiment 1a): Impact of the presence of sperm with abnormal tail morphology (CD, bent tail) on motility of sperm with normal tails

**Figure 1:**
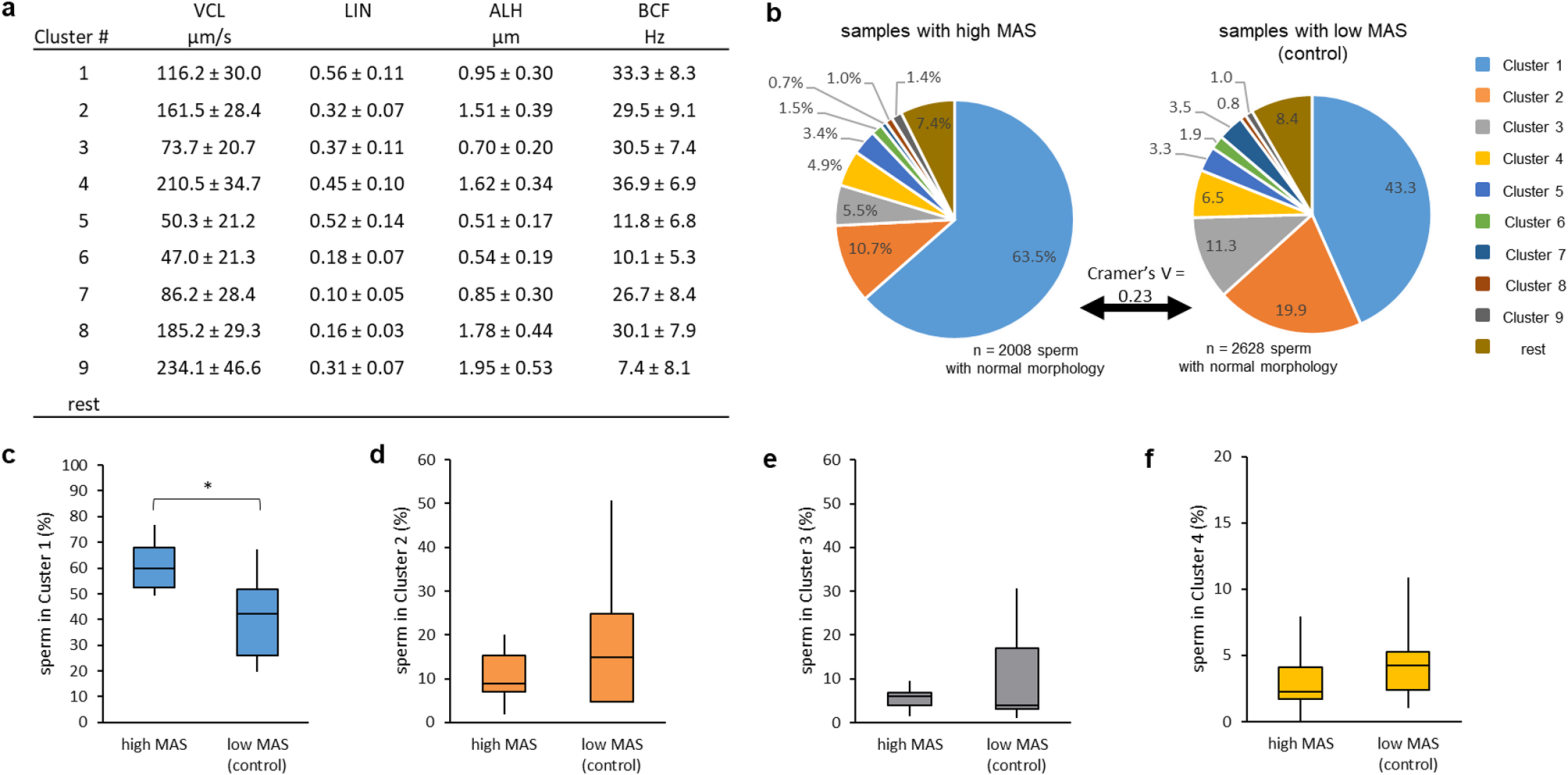
Distribution of morphologically normal spermatozoa originating from extended semen samples with either enhanced (≥ 15% = high MAS; n = 10 boars) or low (< 5% = low MAS; n = 9 boars) prevalence of cytoplasmic droplets and/or bent tails. Data are based on sperm tracks analysed after a 30-min incubation period at 38 °C in Beltsville Thawing Solution. (**a**) Motility descriptors (mean ± SD) are given for each cluster. **b**) Distribution of spermatozoa to the individual cluster based on their origin from high MAS samples or low MAS samples. Cramer’s V indicates a small effect of the sample on sperm distribution. (**c**)–(**e**) Comparison of sperm distribution in high MAS samples (n = 10 boars) and low MAS samples (n = 9 boars) for Cluster 1 (**c**), Cluster 2 (**d**), Cluster 3 (**e**) and Cluster 4 (**f**). Boxes show the interquartile range. Vertical lines indicate minimum and maximum, while the horizontal line in the box indicates the median. An asterisk (*) indicates a significant difference between samples (*P* < 0.05).

The main sperm motility subsets (clusters) for high and low MAS samples after a 30-min incubation period are described in Fig. 1a. The origin of normal spermatozoa had a significant impact on the distribution of the cells to the different motility patterns (Fig. 1b), but the effect size was small (χ^2^ (9; N = 4636) = 241.2, *p* < 0.0001, V = 0.23). Most spermatozoa from both origins showed the same movement pattern, i.e. they were allocated to Cluster 1 (Fig. 1b). However, morphologically normal spermatozoa from samples with high MAS contained on average more sperm from Cluster 1 (Fig. 1c). Overall, sperm with normal morphology from low MAS samples showed fewer differences in the amount of sperm allocated to a specific movement pattern than sperm from high MAS samples (Fig. 1b–f).

#### Experiment 1b) Impact of sperm tail morphology on sperm movement

**Figure 2:**
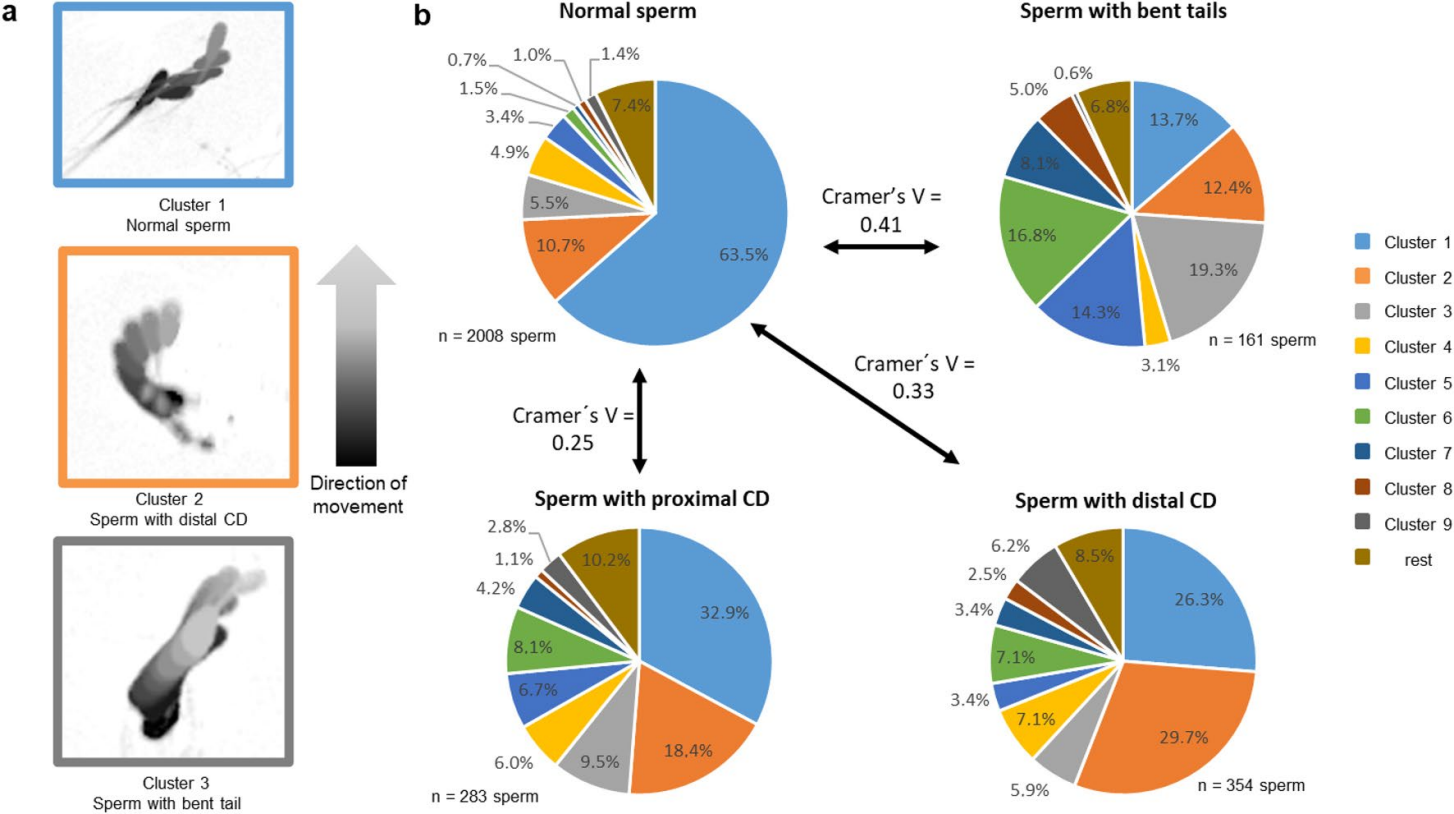
Representative sperm tracks in the three main clusters (**a**) and distribution of spermatozoa with cytoplasmic droplet (CD) or bent tail compared to sperm with normal tail morphology (normal sperm) in nine kinematic clusters (**b**). Data are based on sperm tracks analysed after a 30-min incubation period at 38 °C in Beltsville Thawing Solution from extended semen samples (n = 10 boars) with enhanced prevalence (≥ 15%) of cytoplasmic droplets or bent tails. Only clusters containing at least 5% of sperm in any of the sperm subsets were considered for evaluation. Cramer’s V (range from 0 to 1) indicates the effect size of a certain morphological defect on sperm distribution to the different cluster (movement patterns). V < 0.1 = no effect; 0.1 ≤ V < 0.3 small effect; 0.3 ≤ V < 0.5 moderate effect; V ≥ 0.5 = large effect.

Exemplary track patterns of sperm in the three main clusters (Clusters 1, 2 and 3) are presented in Fig. 2a. In morphological normal sperm, most sperm tracks (63%) were allocated to Cluster 1 with a high LIN, moderate values for VCL and moderate values for ALH. The sperm morphology had a significant impact on the distribution of the cells to the different motility patterns (Fig. 2b). The effect size was moderate for sperm with bent tails (χ^2^ (9; N = 2169) = 362.4, *p* < 0.0001, V = 0.41) and sperm with distal CD (χ^2^ (9; N = 2362) = 251.2, *p* < 0.0001, V = 0.33), but low for sperm with proximal CD (χ^2^ (9; N = 2291) = 144.7, *p* < 0.0001, V = 0.25). The effect in all groups was mainly based on a lower number of spermatozoa in Cluster 1 and a higher number of spermatozoa in Cluster 7, the latter being characterised by low values for VCL and LIN. Spermatozoa with distal CD showed a high frequency of cells (30%) in Cluster 2 represented by high values for VCL and ALH.

### Experiment 2. Sperm response to procaine

#### Experiment 2a): Impact of the presence of sperm with abnormal tail morphology (CD, bent tail) on the movement patterns of sperm with normal tails after stimulation with procaine

**Figure 3:**
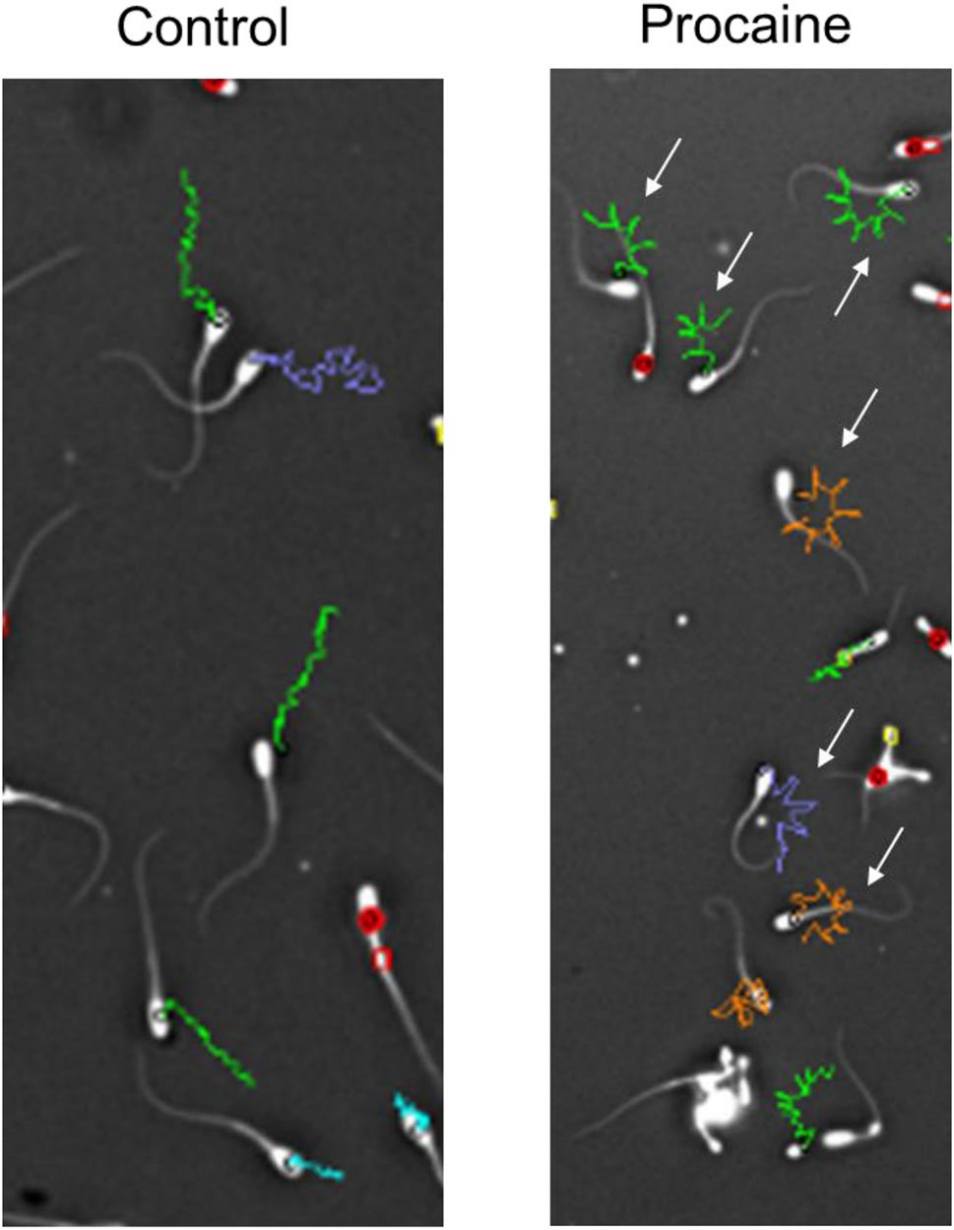
Representative motility tracks of spermatozoa after centrifugation and incubation in non-capacitating Tyrode’s medium without (control) or with procaine. The stimulatory effect of procaine can be inferred from the star-like tracks in the right picture (white arrows). Images are screenshots from computer-assisted semen analysis output.

**Figure 4:**
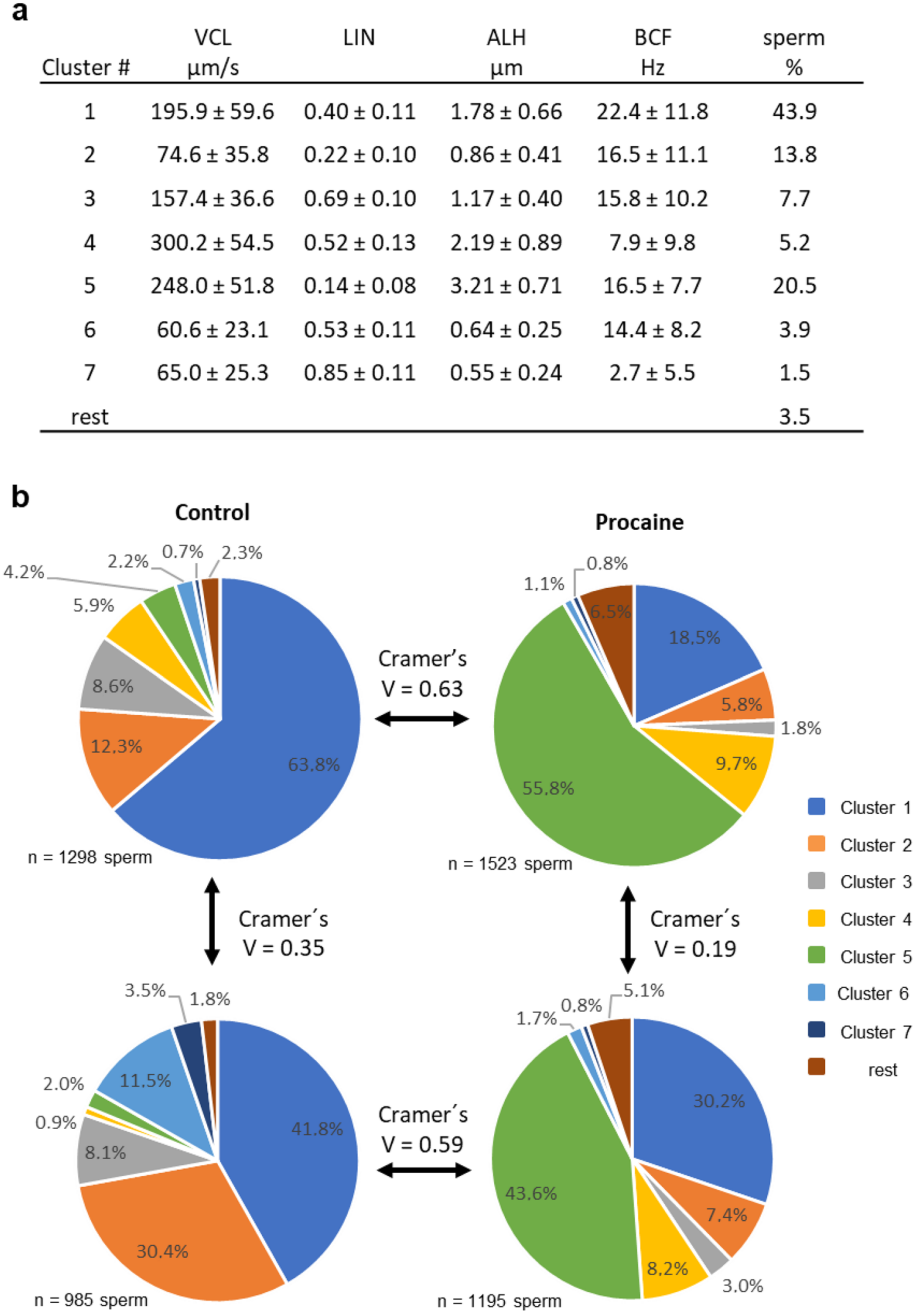
Motility patterns of morphologically normal spermatozoa originating from extended semen samples with either enhanced (≥ 15% = high MAS; n = 10 boars) or low (< 5% = low MAS; n = 9 boars) prevalence of sperm with cytoplasmic droplets and/or bent tails in the absence or presence of procaine. Extended semen samples were centrifuged, and pelleted sperm incubated in non-capacitating Tyrode’s medium without (control) or with 7.3 mM procaine for 10 min at 38 °C. (**a**) Motility characteristics of sperm from the different clusters. Only clusters containing at least 5% of sperm in any of the sperm subsets from this experiment were considered for evaluation. (**b**) Effect of procaine on motility patterns of sperm with normal morphology from samples with low MAS (upper pie charts) or high MAS (lower pie charts). Cramer’s V (range from 0 to 1) indicates the effect size of sample origin or procaine treatment on sperm distribution to the different cluster (movement patterns). V < 0.1 = no effect; 0.1 ≤ V < 0.3 small effect; 0.3 ≤ V < 0.5 moderate effect; V ≥ 0.5 = large effect.

**Figure 5:**
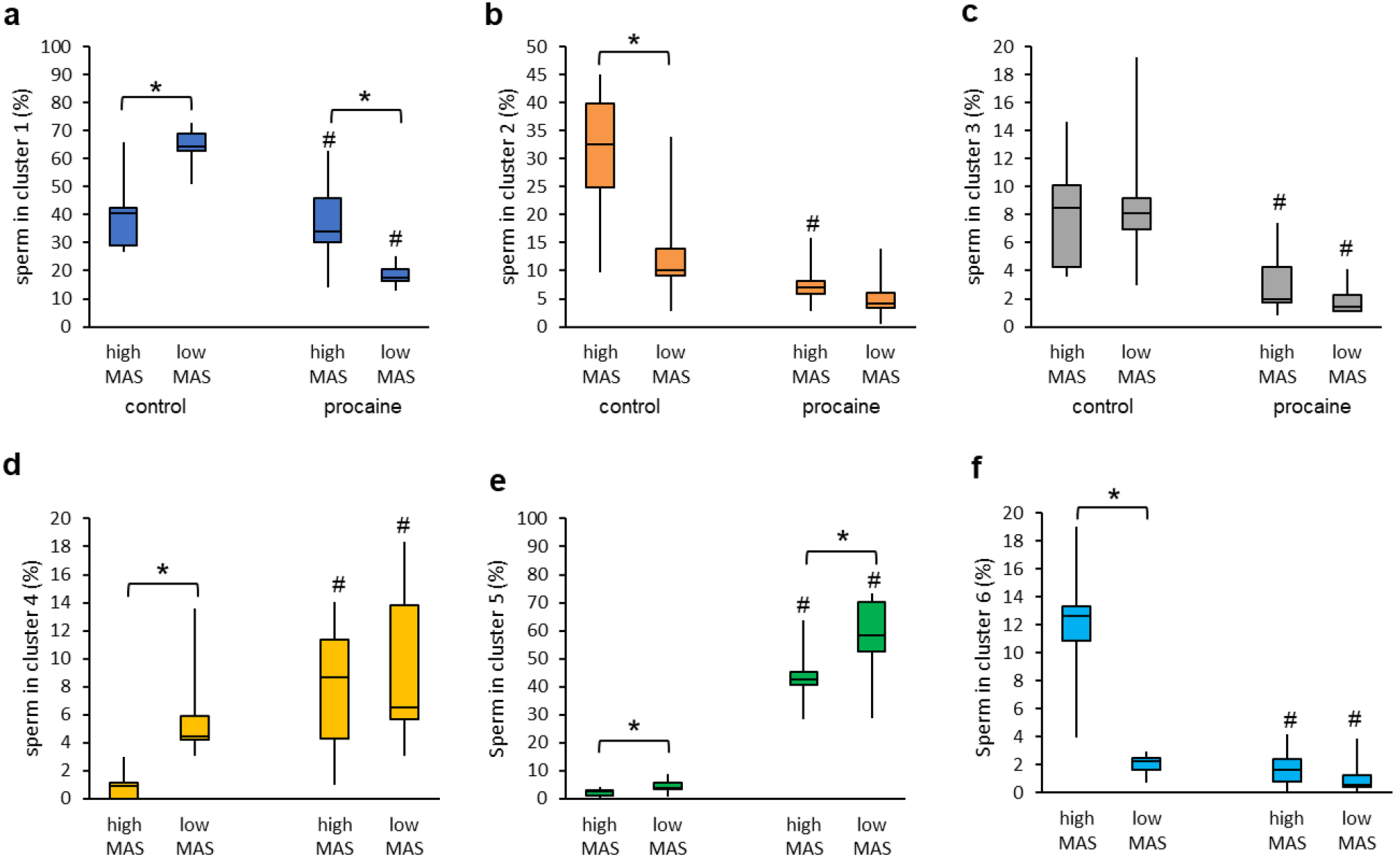
Impact of procaine on motility patterns of morphologically normal spermatozoa originating from extended semen samples with either enhanced (≥ 15% = high MAS; n = 10 boars) or low (< 5% = low MAS; n = 9 boars) prevalence of sperm with cytoplasmic droplets and/or bent tails. Extended semen samples were centrifuged, and pelleted sperm incubated in non-capacitating Tyrode’s medium without (control) or with 7.3 mM procaine for 10 min at 38 °C. Graphs (**a**) to (**f**) show the six major sperm subpopulations as described in Fig. 4. Boxes show the interquartile range. Vertical lines indicate minimum and maximum, while the horizontal line in the box indicates the median. An asterisk (*) indicates a significant difference between high MAS and low MAS samples (*p* < 0.05). A hash (#) indicates a significant difference between the sample treated with procaine and the respective control (*p* < 0.05).

Incubation for 10 min in presence of procaine resulted in a distinct change in the movement pattern in many spermatozoa with normal morphology. The effect of procaine was characterised by star-like motility tracks (Fig. 3) which indicated fast movement with low LIN and high ALH of lateral head-displacement (Fig. 4a, Cluster 5). The origin of the normal spermatozoa from either high or low MAS samples had a moderate effect size under control conditions (χ^2^ (7; N = 2283) = 287.4, *p* < 0.0001, V = 0.35). Nonetheless, exposure to procaine had a significant impact on the motility patterns of the spermatozoa (Figs. 4b and 5) with a strong effect size for normal sperm from both low MAS samples (χ^2^ (7; N = 2920) = 1146.1, *p* < 0.0001, V = 0.63) and high MAS samples (χ^2^ (7; N = 2180) = 761.5, *p* < 0.0001, V = 0.59). In both cases, a significant increase in the average percentage of sperm from Cluster 5 was observed as the main change (Figs. 4b and 5e). Noteworthy, the effect of sperm origin was small after procaine stimulation (Fig. 4b).

#### Experiment 2b): Impact of sperm tail morphology on movement patterns after stimulation with procaine

**Figure 6:**
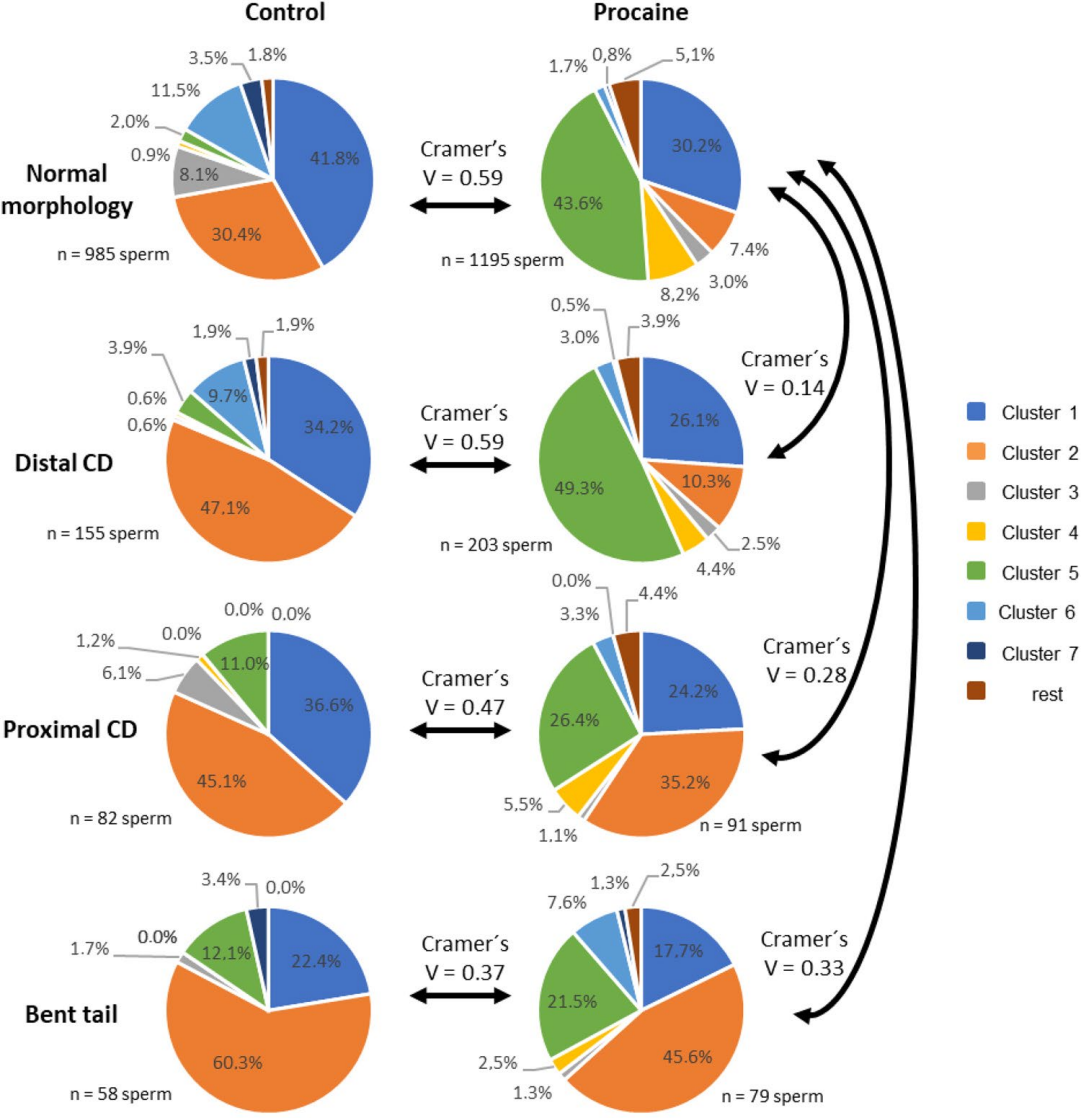
Effect of stimulation with procaine on the distribution of spermatozoa with a cytoplasmic droplet (CD), bent tail or normal tail morphology to clusters which represent different sperm motility patterns. Data are based on sperm tracks from semen samples (n = 10) with ≥ 15% cytoplasmic droplets or bent tails (= high MAS samples). Extended semen samples were centrifuged and incubated in non-capacitating Tyrode’s medium without procaine (control) or with 7.3 mM procaine for 10 min at 38 °C. Only clusters containing at least 5% of sperm in any of the sperm subsets analysed in Figs. 4 and 6 were considered for evaluation. Cramer’s V (range from 0 to 1) indicates the effect size of sperm morphology or procaine treatment on sperm distribution to the different clusters (movement patterns). V < 0.1 = no effect; 0.1 ≤ V < 0.3 small effect; 0.3 ≤ V < 0.5 moderate effect; V ≥ 0.5 = large effect.

After centrifugation and incubation, the differences in the motility patterns between morphologically normal spermatozoa and sperm with proximal or distal CD, or bent tail were moderate to small (data not shown). The effect size of procaine stimulation was moderate for sperm with proximal CD (χ^2^ (7; N = 173) = 37.6, *p* < 0.0001, V = 0.47) and for sperm with bent tail (χ^2^ (7; N = 185) = 18.4, *p* < 0.01, V = 0.37; Fig. 6). For sperm with distal CD, the effect size was strong (χ^2^ (7; N = 358) = 124.1, *p* < 0.0001, V = 0.59) and the effect of the distal CD on the distribution of sperm to the different motility cluster was very small (χ^2^ (7; N = 1825) = 37.7, *p* < 0.0001, V = 0.14) when compared to morphologically normal sperm.

### Experiment 3: Sperm viability.

#### Experiment 3a): Impact of the presence of sperm with abnormal tail morphology (CD, bent tail) on the viability of sperm with normal tails

**Figure 7:**
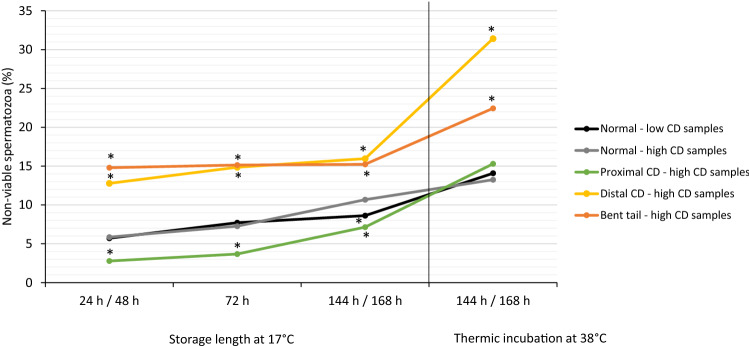
Prevalence of non-viable sperm with normal tail morphology or with proximal or distal CD or bent tail during storage at 17 °C up to 168 h and subsequent thermic incubation at 38 °C for 3 h. Extended semen originated from samples with either enhanced (≥ 15% = high MAS; n = 15 boars) or low (< 5% = low MAS; n = 11 boars) prevalence of cytoplasmic droplets and/or bent tails. An asterisk (*) indicates a significant difference between sperm with a CD or bent tail and normal sperm within a time point (*p* < 0.05). There were no differences between normal sperm from high and low MAS samples (*p* > 0.05).

Sperm morphology had a significant impact on viability. In sperm groups with distal CD or bent tails, percentages of sperm with a defective plasma membrane (non-viable sperm) were higher (*p* < 0.05) compared to the group of morphologically normal sperm at all storage time points and after thermic stress. The group of sperm with proximal CD contained a slightly lower percentage (*p* < 0.05) of non-viable sperm compared to the group of normal sperm (Fig. 7).

#### Experiment 3b): Impact of sperm tail morphology on sperm viability

The origin of the normal spermatozoa from high or low MAS samples had no effect on sperm viability during semen storage and after thermic stress (Fig. 7). Among normal sperm, there was only a moderate (*p* < 0.05) increase in non-viable sperm from 24 h (low MAS samples: 5.7%; high MAS samples: 5.9%) until 168 h of storage (low MAS samples: 8.6%; high MAS samples: 10.7%).

## Discussion

In this study, single cell observations of sperm kinematics and membrane stability open the perspective to compensate a higher prevalence of CD in boar semen for a higher number of spermatozoa in the semen dose. This seems to be possible because deficiencies were only detected in sperm with CD (and/or bent tails) but not in normal sperm of semen samples with an enhanced prevalence of this malformation.

Functional deficiencies in spermatozoa with CD were detected by cluster analyses of sperm motility traits and the ability to maintain viability under storage and thermic stress conditions. The higher prevalence for a reduced linear movement pattern in spermatozoa with retained CD can be related to their immature state, in analogy to epididymal spermatozoa, which acquire their capacity for progressive and vigorous forward motility during transit from the caput to cauda epididymis^28,29^. The sperm’s ability to activate its motility (forward and hyperactivated) and to capacitate belongs to the principal characteristics of a ‘mature’ spermatozoon^30^. Spermatozoa with a proximal CD could be considered less mature compared to sperm with a distal CD, which in the present study is mirrored in the lower response to the pharmaceutical activator procaine.

Regarding the methodology, an unsupervised clustering approach was used on the raw kinematic data from each single motile spermatozoon. In this way, subjective classification by manufacture-defined subcategories with respect to progressiveness, speed or directionality was avoided. The adequateness of our explorative statistical approach is reflected in cluster solutions which explain a large amount of the variability in the single sperm data set and still can be biologically interpreted^18^. Although data analysis obtained from comparable samples by different CASA systems could yield slightly different quantitative cluster solutions in terms of number and sizes of main clusters, it is expected to find similar qualitative relationships between sperm morphology and movement patterns as reported here.

The observation that the kinematic response to procaine of spermatozoa with distal CD is similar to normal spermatozoa might indicate that distal CD have only minor relevance for fertility. On the contrary, plasma membranes of spermatozoa with distal CD reacted more sensitively to long-term storage and thermic stress. A high degree of membrane destabilisation and concomitant loss of in vitro capacitation competence in semen samples with a higher incidence of sperm with a CD was also previously reported^21^, though without distinguishing between proximal and distal CD. Plasma membrane properties develop during epididymal transit with the sequential addition of glycoproteins^29^, modifications of protein phosphorylation^31^ and uptake of organic osmolytes^32^ and other components from the epididymal secretions. Studies on the epididymal proteome suggest that sperm maturation progresses towards preservation of sperm viability^29^. In this view, it was unexpected that sperm viability in the presence of distal CD was lower compared to less matured spermatozoa characterised by a proximal CD. However, comparisons with studies on epididymal spermatozoa might be misleading because the remodelling of the boar sperm proteome during ejaculation is ignored. It has been suggested that the heterogeneity of protein composition of ejaculated boar spermatozoa could be related to a variability in sperm morphology^33^. Alternatively, the inability of CD to migrate along the midpiece and/or to be shed upon contact with the seminal plasma could be caused by defective spermiogenesis or dysfunction of accessory glands rather than disturbed epididymal maturation^14^. In these causative scenarios, response of CD-bearing spermatozoa to the epididymal environment would be disturbed independently of their maturation stage.

In this study, sperm with bent tails were considered as a positive control in the kinematic studies due to their inherent disability for progressive, linear movement. Interestingly, such spermatozoa displayed a reduced viability similar to those with distal CD. The majority (63%) of bent tails in this study were classified as “distal midpiece reflex” defined as the presence of a CD in a 180° bend in the sperm tail^3^, a defect which is less common in boars compared to bulls where it presents the most frequent sperm tail abnormality^34^. Whether the reduced viability was associated with the CD was not clarified in the present experiments and is also of minor relevance for the aims of this study. Spermatozoa with bent tails, regardless of the presence of a CD, may not reach the site of fertilisation. The exclusion of sperm from interaction with the oocytes is less clear in spermatozoa with CD but otherwise regular morphology of the flagellum. The presence of CD per se does not hinder fertilisation as shown by fertility obtained with boar spermatozoa from different regions of the epididymis^35^. Nonetheless, as previously mentioned, an enhanced incidence of spermatozoa with a retained CD in ejaculated semen clearly reduces fertilisation chances.

If a high prevalence of spermatozoa with a retained CD is indicative of disturbed spermiogenesis, also morphologically normal appearing spermatozoa could be affected by testicular dysfunction. Moreover, it was speculated that reactive oxygene species produced by CD would cause functional deficiencies also in normal spermatozoa^17^. In these cases, chances for compensation would be reduced. In the single cell approach used here, we demonstrated that most normal spermatozoa in semen samples with a high incidence of sperm with a CD do not differ in kinematics and viability from normal spermatozoa in samples with a low incidence of spermatozoa with a CD. These observations indicate the possibility that sufficiently morphologically normal spermatozoa could outweigh a sperm subpopulation with CD in a semen dose. Still, it cannot completely be ruled out that normal spermatozoa in high MAS samples suffer from other functional deficiencies which remained undetected by the investigations performed here. However, an earlier microscopic study by Flesch, et al. ^36^ supports the view that specifically the subpopulation of sperm with CD are affected by dysfunction, as shown in their lack of response to the capacitation trigger bicarbonate, probably caused by a disturbed cholesterol organisation in the membrane lipid bilayer.

Noteworthy, in the present study mimicking the most common situation in AI practice, semen samples from sexually mature, healthy, fertile AI boars with a temporarily moderately enhanced incidence of CD were used. Compensation of this defect should only be considered under these circumstances. In contrast, a permanent or a temporary incidence of a high sperm population with CD could be indicative of a severe testicular or epididymal dysfunction which might be less compensable. The necessary increase in sperm numbers in samples with CD to reach the full fertility potential in sow herds should consider minimum standards^1^ for motility and other morphological abnormalities. Fertility traits show a curvilinear response to sperm dose^11,37^. The extent of compensation depends on the sperm dose of the regular AI dose after onset of the asymptotic part of the dose–response fertility curve. Taking into account current standards for semen use for artificial insemination^1^, compensation for the presence of CD between 15 and 25% should be eligible. This notion is supported by recent retrospective analysis of field data showing no difference in fertility between semen samples from young boars with low (< 5%) and enhanced (≥ 15%) prevalence of spermatozoa with a CD^38^.

In conclusion, the present single cell study indicates that transiently moderately enhanced prevalence of spermatozoa with a CD can be compensated for maintenance of fertility by an increase in morphologically normal spermatozoa in the porcine semen dose. To ensure high fertility, minimum semen requirements for other morphological traits and motility should be fulfilled. Compensation for CD would allow AI centres to provide the requested genetics, especially in the late summer season where this sperm abnormality is typically increased in boars.

## Data Availability

All data generated or analysed during this study are available from the corresponding author on reasonable request.
